# Antimicrobial Use in Brazilian Swine Herds: Assessment of Use and Reduction Examples

**DOI:** 10.3390/microorganisms9040881

**Published:** 2021-04-20

**Authors:** Mauricio Cabral Dutra, Luisa Zanolli Moreno, Ricardo Augusto Dias, Andrea Micke Moreno

**Affiliations:** Department of Preventive Veterinary Medicine and Animal Health, School of Veterinary Medicine and Animal Science, University of São Paulo, Av. Prof. Dr. Orlando Marques de Paiva 87, São Paulo 05508 270, SP, Brazil; maucdutra@hotmail.com (M.C.D.); luzanolli@gmail.com (L.Z.M.); ricardodias@usp.br (R.A.D.)

**Keywords:** swine, antimicrobial use, biosecurity, one health, disease prevention

## Abstract

Brazil, as a major pig producer, is currently experiencing the widespread use of antimicrobials as a serious issue to be addressed. For measures to be taken in this direction, the extent of the problem must be known. The goal of this study was to evaluate the use of antimicrobials in 25 Brazilian swine herds. Antimicrobial use from birth to slaughter was correlated with biosecurity and productivity. After the first assessment (2016; M0), 13 herds implemented good practices to reduce antimicrobial use. Four years after the implementation of these measures (2020; M1), data about antimicrobial usage from these herds were collected. The results of the first assessment (M0) demonstrated a troublesome scenario: the mean value of antimicrobials used was 358.4 mg/kg of pig produced; the median of the pig’s lifetime exposure to antimicrobials was 73.7%, and the median number of drugs used was seven. A positive correlation between the antimicrobials consumed and the pig’s antimicrobial exposure time was detected. Nevertheless, these data did not correlate with biosecurity score or productivity. A significant difference was detected in M1, where a median 30% reduction in antimicrobials consumed was detected. There was also a 44.3% reduction of the pig’s lifetime exposure to antimicrobials. The median number of drugs used was reduced from seven to five. Antimicrobial use did not always reflect the sanitary condition or the real therapeutic needs, easily leading to overuse.

## 1. Introduction

Large amounts of antimicrobials have been used for disease prevention and control in intensive food animal production. In some countries, antimicrobial agents are considered a management tool. In many cases, farmers, technicians, and veterinarians might not even be aware that they are using them for this purpose. Excessive and continued antimicrobial use provides conditions for the selection, spread, and persistence of antimicrobial-resistant bacteria. These bacteria are capable of causing infections in animals and humans [1]. Antimicrobial resistance is a global threat to human and animal health, as the loss of antimicrobial efficacy compromises the clinical treatments used [2].

Brazil is the fourth-largest producer and fourth-largest exporter of pork in the world with a swine population of 2,017,645. From 3983 million tons of pork produced in 2019, a total of 81% was consumed in the domestic market, with an average consumption of 15.3 kg of pig meat per inhabitant, while the remaining 19% was exported to different countries [3]. Antimicrobial use in swine for prophylactic and metaphylactic purposes is not prohibited or controlled in Brazil. Some antimicrobials are allowed as growth promoters in specific livestock species. Other active molecules, however, were banned from use in animal production for this purpose. This was per the Normative Instructions published over the last two decades by the Brazilian government. When this study started, some antimicrobials were permitted to be used as growth promoters in swine. They included avilamycin, bacitracin, enramycin, flavomycin, halquinol, lincomycin, salinomycin, tiamulin, tylosin, and virginiamycin. The use of colistin as a growth promoter was prohibited in November 2016, but therapeutic, prophylactic, and metaphylactic use is still permitted [4]. In January 2020, lincomycin, tiamulin, and tylosin were also prohibited as growth promoters in Brazil [5].

The Brazilian government has been working since 2018 on the implementation of The National Action Plan on Antimicrobial Resistance in Agriculture. The plan’s goals include health education, epidemiological studies, surveillance, and monitoring of antimicrobial use. It also aims to strengthen biosecurity and implement control measures and promote rational antimicrobial use in livestock. Nevertheless, as of this writing, no official data on the volume of antimicrobials used in livestock production is publicly available. The existing data is provided by the National Union of Animal Health Products Industries (SINDAN). It includes the sale of antibiotics for both livestock and small animals, without discrimination among species [4].

Antimicrobial use in swine in Brazil has increased in the last 20 years, according to the perception of veterinarians and agroindustry professionals. The reason for this growth is particularly complex, and includes the greater availability of antimicrobials, fear of disease outbreaks, increased market competitiveness, lack of technical guidance and especially lack of a legal control regarding antimicrobial usage in livestock. At the same time, there was as increase in antimicrobial resistance rates in different bacterial species isolated from swine at diagnostic laboratories. There were also reports of bacteria carrying resistance genes against antimicrobials important to human health. Some examples are descriptions of *Escherichia coli* carrying CTX-M-15, CMY-2, MCR-1, or MCR-3 [6,7,8], *Salmonella* Typhimurium carrying qnrE1 [9], methicillin-resistant *Staphylococcus aureus* ST398 [10], and *Enterococcus faecalis* resistant to oxazolidinones carrying *cfr* and *optr*A genes [11], which were isolated from Brazilian swine samples.

Considering the lack of information about the pattern and amount of antimicrobials used in Brazilian swine production, in this study, we evaluated antimicrobial use as a preventive or growth promoter in 25 swine herds located in the country’s main swine-producing states. The antimicrobial usage was correlated with the biosecurity score and productivity index of studied herds. The use of antimicrobials was reevaluated four years after the first assessment in 13 of the initially selected herds. The implementation of a good management practices program was assessed in these 13 production systems, and the observed results were discussed.

## 2. Materials and Methods

### 2.1. Selection of Herds, Visits, and Data Collection

A convenience sample of 25 pig herds was approached with an invitation to participate in the study. Herds included a minimum of 150 and a maximum of 15,000 sows and originated from nine Brazilian states. Data on production parameters, antimicrobial usage, biosecurity practices, vaccination programs, and other management practices were collected. This study was approved by the FMVZ-USP ethics committee number 5446170717.

All swine herds visited are independent producers who provide animals for slaughter in the Brazilian domestic market. But they are representative of the national swine chain management and technical levels. The main characteristics of the evaluated herds are presented in Table 1. All herds were negative for porcine reproductive and respiratory syndrome virus (PRRSV). In addition, status to *Mycoplasma hyopneumoniae* was reported. Swine herds were classified as farrow-to-finish herds (with all animals in the same site), piglet production unit and finishing herds (with animals in two different sites), or only piglet production unit.

From January to December 2016, the 25 herds were submitted to at least one visit to collect all the data described above. This was called moment zero (M0). After the first assessment, 13 herds accepted the implementation of good practices and measures to reduce antimicrobial usage. The main researcher monitored these herds. From June to November 2020, they were submitted to a new data collection, called moment 1 (M1).

### 2.2. Productivity and Antimicrobial Use Data Collection

The productivity and antimicrobials usage data were obtained through personal interviews with the owner and/or herd manager. All data were collected by the same veterinarian/researcher in all herds. The herd’s productivity was evaluated by calculating the average kg of pig produced by a sow per year.

Antimicrobial usage was evaluated considering their use as a growth promoter, and preventive or metaphylactic programs involving large groups of animals, including injectable use (in suckling piglets only), feed use (in most cases), or water use (eventual).

Calculations considered milligrams of antimicrobials per kilogram of pig produced in each herd and the period of use (mg/kg biomass) as described in [12]. The following age categories were used for the calculation: suckling piglets (birth to an approximate weight of 6 kg—weaning), weaners (weaning to an approximate weight of 30 kg), fatteners (~30 kg to slaughter), and adult pigs. The kg animal at risk is the total weight of pigs for that age category (in kilograms).

### 2.3. Biosecurity Data Collection

Qualitative and quantitative data regarding the herd’s health status, management, structure, biosafety, and vaccines used were evaluated. The applied questionnaire (Supplementary Material) is based on the American Association of Swine Veterinarians [13], and the Production Animal Disease Risk Assessment Program (PADRAP). This survey is widely used by swine practitioners in North America, and the information obtained can be adapted for more generalized purposes [14]. A strict protocol was used to visit, interview, and collect the data in the participating herds, guaranteeing a similar data collection and entry. To minimize variations, only one veterinarian/researcher who knew all the producers conducted the visits and interviews. Data on the environmental structure and condition of the herd were visually assessed by the veterinarian/researcher.

Briefly, the questionnaire contains 120 questions divided into 16 areas. The questionnaire addresses herd susceptibility to external and internal risks. These risks are related to introducing exotic agents to the system, and the dissemination of agents already present on the property, respectively. Each item/question was classified as “suitable” (implemented and followed appropriately), representing 10 points; “requiring adjustments” (implemented but requiring corrections), worth 5 points; and “inadequate” (not yet implemented), with zero points. The maximum score that could be reached is 1200, and the higher this value, the better the observed biosecurity conditions.

### 2.4. Measures to Reduce Antimicrobial Use

Between the two data collections (M0 and M1), in the 13 herds in the study, a series of management improvements were implemented. Their focus was on reducing the use of antimicrobials. These measures are described in Supplementary Material. The main researcher visited these herds at least three times a year during this period.

### 2.5. Data Analysis

Data management was performed initially in Microsoft Excel 2010. The statistical analyses were performed with SPSS 16.0 (SPSS, Inc., Chicago, IL, USA) and the level of significance of *p* ≤ 0.05 was adopted. Quantitative variables’ normality was assessed by the Shapiro–Wilk test. The correlations between the antimicrobial usage with the biosecurity score and productivity index of studied herds were performed using Spearman’s rank correlation. The Wilcoxon test for paired samples was applied for the comparison of M0 and M1 data.

## 3. Results

**Figure 1:**
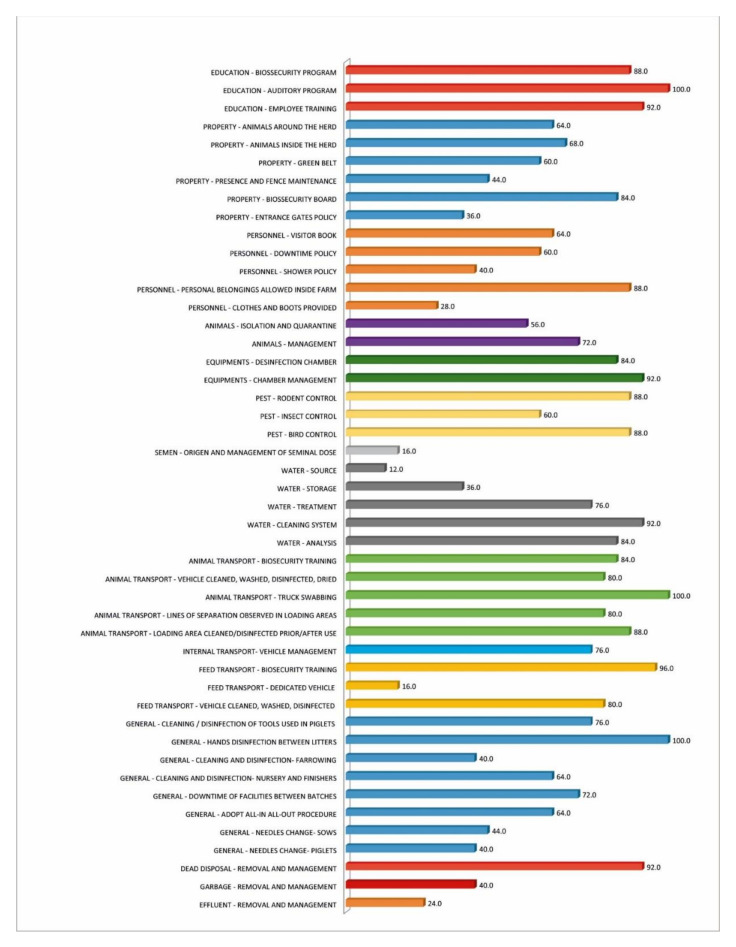
Percentage of farms requiring adjustments according to main biosecurity aspects (M0).

Table 1 presents the results for the biosecurity score and the herd’s characterization. The productivity of herds had an average of 2852 kg pig/sow/year. This index presented a large variation from 2004 to 3979 kg (Table 2). The average biosecurity score obtained for the studied herds was 708 points, ranging from 435 to 1105 points. Figure 1 presents a resume of the internal and external biosecurity conditions of the 25 herds and the main points requiring adjustments.

Considering antimicrobial use, we observed an average of 358.4 mg/kg of pig produced. This value varied from 5.4 to 585.6 mg/kg among the 25 herds (M0). The pig’s lifetime antimicrobial exposure was a median of 73.7%, with a variation of 2.9% to 90.4% of the time (from birth to slaughter). During this period, the animals had contact with two to 11 different antimicrobials (average 7 drugs) (Table 2). A total of 26 drugs from 14 antimicrobial classes were used in these 25 herds (Table 3).

**Figure 2:**
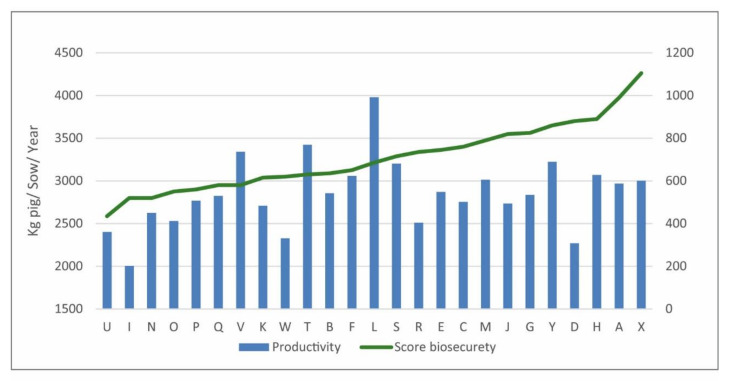
Productivity parameters according to biosecurity score classes identified in 25 herds (M0).

When the antimicrobial use was correlated with the productivity index, no statistical correlation was detected (r= −0.083, *p* = 0.693). However, in Figure S1, it is possible to observe that the group of herds presenting the lower antimicrobial use tended to present higher productivity. The biosecurity score also did not present a statistical correlation with the amount of antimicrobial used (r= −0.058, *p* = 0.783). Even though herds with higher biosecurity scores tended to present a better productivity index (Figure 2), there was no statistical correlation (r = 0.348, *p* = 0.088). The positivity for *Mycoplasma hyopneumoniae* also did not correlate with the amount of antimicrobials used in the studied herds (r = −0.312, *p* = 0.129).

**Figure 3:**
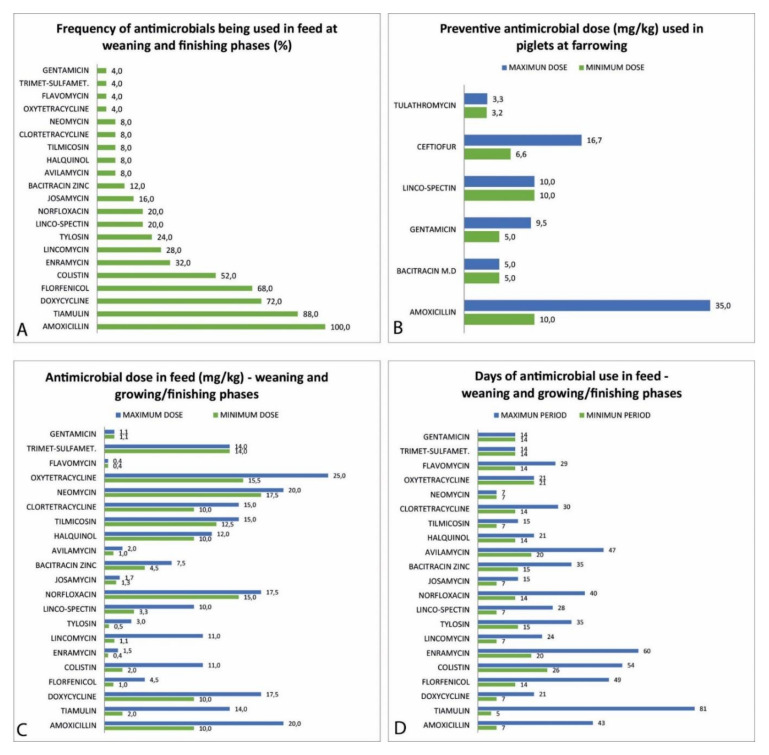
Antimicrobial usage data from evaluated herds. (**A**) Percentage of herds using different drugs in feed in the weaning and growing/finishing phases (%). (**B**) Percentage of herds using different drugs in piglets at farrowing. (**C**) Antimicrobial dosages used in preventive protocols in weaning and growing-finishing pigs (mg/kg of weight) at evaluated herds at M0. (**D**) Maximum and minimum period of treatment in days to each drug administered in-feed at the weaning and growing/finishing phases.

Among the 25 herds, 18 (72%) presented preventive antimicrobial use in suckling piglets with one or more drugs. Fifty percent of these herds used more than one drug at this phase. The drugs more often used were ceftiofur (40%), followed by amoxicillin (24%), gentamicin (16%), lincomycin/spectinomycin (8%), tulathromycin (8%), and bacitracin methylene disalicylate (4%). This data is presented in Figure 3A. Table 3 shows this antimicrobial use and the drugs’ use as preventive protocols in the weaning and growing-finishing phases.

The frequency of antimicrobials used in the weaning and growing-finishing phases is demonstrated in Figure 3B. Amoxicillin use was detected in all studied herds (100%), followed by tiamulin (88%), doxycycline (72%), florfenicol (68%), and colistin (52%). The dosage of antimicrobials used in these phases presented a large variation that is shown in Figure 3C. Colistin, lincomycin, combination lincomycin/spectinomycin, tiamulin, and tylosin were used in therapeutic doses or as growth promoters in some situations. This explains the large variation observed.

The period of treatment presented a wide variation among the 25 herds in suckling piglets and the weaning/growing-finishing phases. Details about this data are shown in Figure 3D. The drugs with a greater range of days of use were tiamulin (5 to 81 days), enramycin (20 to 60 days), amoxicillin (7 to 43 days), florfenicol (14 to 43 days), and colistin (26 to 54 days). Only four drugs were used with the same prescription times: gentamicin, neomycin, oxytetracycline, and trimethoprim-sulfamethoxazole.

Paired data about antimicrobial usage in the 13 study herds were collected (Table 2 and Figure S2) four years after a good practices program to reduce antimicrobial usage. A significant difference was detected comparing M0 and M1 data for mg of antimicrobials per kilogram of pig produced, the number of antimicrobials used, pig’s lifetime exposed to antimicrobials, and productivity (*p* = 0.034, 0.025, 0.015, and 0.019, respectively). We observed that 10 herds presented a reduction in the number of antimicrobials used. One was stable with a small use, and two presented increased usage. The reduction varied from 16% to 94.2%, with a median 30% reduction in antimicrobials consumed per kg of pig produced. Considering the lifetime exposure of pigs to antimicrobials, nine herds presented a reduction since the first assessment. An increase was observed in only two herds. The variation of reduction was from 42.9% to 78.4%. The number of drugs used reduced in nine herds, increased in two, and maintained the same in two herds. The median number of drugs used was reduced to five. The median productivity in these 13 herds increased from 2891 to 3296 kg pig/sow/year, an increase of 14%.

## 4. Discussion

Currently, in intensive pig production systems in Brazil, antimicrobials are often administered regularly by farmers. This is sometimes under the direction of a veterinarian, but without requiring a veterinary prescription [4]. Therefore, pig farmers or farm managers play a crucial role in the administration of antimicrobials to pigs. As a result, valid data on the actual dose and group treatment are the most standardized and well known by these persons [15]. This is the primary reason study data collection was restricted to a standardized group level of preventive treatments and the use of growth promoters.

The trust relationship between the producers and the main researcher and free access to the farm and data was important in choosing these 25 herds. The decision to work with a small group of farms was also important, but with reliable data. The herds evaluated are located in the three largest pig-producing regions in Brazil (Southeast, South, and Midwest), with a total of 61,390 sows. This is very representative of our reality, although it is not a statistically significant sample considering the size of the Brazilian swine population.

Biosecurity level, productivity, and positivity to *M. hyopneumoniae* did not present a statistical correlation with the antimicrobial amount used in the 25 studied herds. Despite this result, Laanan et al. [16] described a positive association between improved biosecurity and reduction in antimicrobial treatments for Belgian farrow-to-finish herds. This was mainly considering the internal biosecurity scores. In this study, we could also observe some examples showing it is possible to achieve high productivity with low antimicrobial use, such as herd L that presented lower antimicrobial usage (5.4 mg/kg) and higher productivity (3979 kg pig/sow/year at M0 and 4260 kg pig/sow/year at M1). At the same time, the lower productivity systems tended to present higher antimicrobial use. This was usually an attempt to compensate for some management or structural fails.

**Figure 4:**
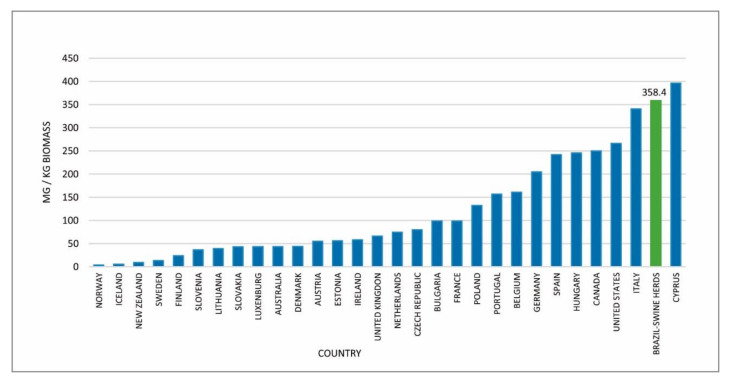
The median antimicrobial amount used in 25 herds of swine evaluated in Brazil compared with the amount of antimicrobials used in different countries during 2012 in food animals (mg/kg of biomass) as described by [17].

Comparing the median amount of antimicrobials used from birth to slaughter in the 25 herds in M0 (358.4 mg/kg) with the antimicrobial amounts used in food animals described by Hillerton et al. [17] in 30 countries, it is possible to verify that the use in swine in this study was inferior only to the one described in Cyprus (Figure 4). These data refer to several animal species produced in these countries. So the fact that the average observed in the 25 Brazilian production systems was so high is troubling. Here, we considered the fixed programs that include preventive antimicrobial use and growth promoters. However, the possible use of injectable drugs for the treatment of sick animals was not included. We also did not consider the treatment of adult animals (boars and sows). This means that the values in the studied herds could even be higher.

The pigs’ lifetime (from birth to slaughter) exposure to antimicrobial is not easy to compare with similar studies. Nevertheless, the observed values seem to be worrisome considering that, in 12 herds, this percentage was greater than 80% of their lifetime. The high number of various drugs used during the pig’s lifetime is also a subject scarcely mentioned in recent literature.

The use of preventive parenteral antimicrobials in suckling piglets in 72% of the studied herds is very expressive. The most frequently used drug in this phase was ceftiofur (40%), followed by amoxicillin (24%). Considering these herds, 50% used more than one parenteral drug as a preventive in suckling piglets. Farmers usually claim that high antimicrobial use in young piglets is to avoid infections during the lactation period (arthritis, omphalitis, meningitis, clostridial infections) and prevent later disease problems. However, a deteriorating effect may be related to antimicrobial use at a young age on the bacterial composition of the gut or respiratory tract. This could result in higher disease susceptibility and an increase of treatment requirements at the later stages of pigs’ lives [15].

The antimicrobials used in the weaning and growing phases presented a large variation. But the antimicrobial most frequently used was amoxicillin, which appeared in 100% of the studied herds. This usage pattern corroborates the findings of [18], who found aminopenicillins in a high number of studies describing antimicrobial use in swine in Germany, Sweden, and Canada. The second antimicrobial most frequently used, tiamulin, was not often used outside. Tiamulin is not described as the most used in several studies, and in our case, it was also used for longer periods. The period of use varied from five to 81 days as described in Figure 3D. We observed that 88% of herds used tiamulin in weaning or growing/finishing pigs. This use can be associated with *M. hyopneumoniae* infection prevention in positive herds. It can also be associated with the prevention of intestinal infections related to *Lawsonia intracellularis* or *Brachyspyra spp*. The tetracycline class was very frequent in our herds, considering the three active antimicrobials in this class (doxycycline, chlortetracycline, and oxytetracycline). This is also common in other countries [18].

The large variation of dose and period of use observed for some drugs suggest that off-label usage is a common practice in these herds. A Belgian survey that quantified antimicrobial use in pigs found several differences among oral and injectable antimicrobials used in off-label group treatments in different herds. For example, overall, 50–75% of the oral formulations were underdosed. From the four most frequently used antimicrobials, doxycycline overdosed in 50–75% of the cases. On the other hand, trimethoprim-sulfonamide was underdosed in 50–75% of the cases. Amoxicillin and colistin were underdosed in 50% and 90% of the cases, respectively [19].

We observed the use of some critically important antimicrobials used in humans, like gentamicin, colistin, fosfomycin, third-generation cephalosporin, and fluoroquinolones, in the studied herds for preventive animal treatment. The usage of these drugs in swine was also reported in different European countries by [18]. However, these drugs must be avoided, and the first treatment choice must be the drugs with low importance in human health. The use of these critical antimicrobials increases the risk of resistance development inside the herd. It has also been reported that the frequency of bacteria carrying antimicrobial resistance genes is high in pig manure and around swine farms. It is estimated that about 75% of the administered antibiotics are not absorbed by animals but are excreted via their feces or urine [20]. This finding is even more pronounced with drugs with low absorption in the gastrointestinal tract like colistin or aminoglycosides [21].

The individualized assessment of the external risks present in the applied biosecurity questionnaire revealed several opportunities for improvement, considering that 56.0% of the herds did not have quarantines or adequately manage them. At least 60.0% of the farmers did not promote adequate pest control, 76.0% did not promote adequate animal water treatment, and 80.0% neglected health risks when transporting animals. The same individualized analysis of internal risks showed negligence in cleaning and disinfection programs in at least 40.0% of the evaluated systems, inadequate use of the facilities, with the “all-in/all-out” system not being adopted by 64.0% of the properties, as well as the downtime of facilities not being respected in 72.0% of the cases. Negligence in adopting measures to minimize the external and internal production systems risks tends to increase the presence of illnesses in the medium and long term. This promotes a greater environmental pressure of infection, ultimately increasing the preventive use of antimicrobials [22].

Among the 13 herds agreeing to improve their management practices and biosecurity (internal and external) controls, the implementation of the proposed measures was not equal. The observed results were not homogeneous. The conditions of facilities, staff capacity, and other factors affected the success of the proposed measures. Considering this fact, we saw some optimal results with a large reduction in the use of antimicrobials and improved productivity, like herd H or W, for example. Some herds, like herd L, which was negative to *M. hyopneumoniae*, kept a low antimicrobial use and increased herd productivity during this period. Two herds showed an increase in antimicrobial usage (D and P). In these herds, the implementation of measures could not be completed due to some intrinsic characteristics of these production systems. Herd D, for example, presents a large number of animals (15,000 sows) making management changes exceedingly difficult.

During the study period, no outbreaks of unexpected diseases occurred in these herds, but the decision to use antimicrobials was not controlled by the research team. Health decisions were always made by the person responsible for the farm. However, we saw that the reduction of antimicrobial use occurred in most of the herds at a significant level. We also found a reduction of exposure time and the number of different drugs used, with an increase of productivity in 10 of the 13 herds.

The implementation of guidelines for the prudent use of antimicrobials in veterinary medicine in Germany presented a significant reduction of 73.0% in their antimicrobial consumption after two years. There was also a reduction from 31.6 to 13.6 days in the treatment period [23]. Similarly, in the Netherlands, the use of antimicrobials in animal production was reduced by 56.0% between 2007 and 2012 [24]. This was the result of intensive collaboration between government officials, veterinarians, and producers, combined with compulsory and voluntary actions with well-defined objectives.

The main reason for the wide use of antimicrobials is the excessive preventive use. Although there was no well-founded justification for the repeated use of preventive group treatments, and despite the associated costs, farmers at large production systems often considered preventive antimicrobial use necessary. Seeking out to observe lower disease rates, lower mortality, and better production results, they also believed to be easier and less labor-intensive to implement preventive medication than treatment of clinically diseased animals after losses have occurred.

In Brazilian systems, it was also possible to verify the existence of a cultural search for maximum productive efficiency that promotes the excessive prophylactic use of antimicrobials and other additives, often disregarding the realization of laboratory diagnosis as an auxiliary measure for making decisions about sanitary programs [15]. Other motivations to antimicrobial usage include the real risk and fear to suffer with bacterial outbreaks; low investments in hygiene, good practices, and biosecurity; and the marketing pressures from pharmaceutical companies.

After the first assessment of the 25 herds and evaluation of results, all herds were contacted. Part of this data was presented to the herd staff, government agents, veterinarians, opinion leaders, and in local symposia and events about antimicrobial usage in swine. The information generated great interest from professionals in the area and actions to reduce antimicrobial use is emerging in different states. New studies are also being developed around the country. This is an opportune time to start a program to reduce the use of antimicrobials with the participation of all members of society and the production chain.

## 5. Conclusions

The main conclusion of this study is that preventive use of antimicrobial did not always reflect the sanitary condition or the real therapeutic needs, easily leading to overuse in most part of the evaluated swine herds. This condition can only be improved with awareness of producers, establishment of good management practices, and implementation of a national program to reduce antimicrobial usage with the participation of all members of the production chain.

## Figures and Tables

**Table 1:** Characteristics of the evaluated swine herds according to state, herd size, *Mycoplasma hyopneumoniae* positivity, and biosecurity level.

Herd	State	Number of Sows	Production System	Status to *M. hyopneumoniae*	Biosecurity Score
A	DF	3500	Two site herds *	Negative	990
B	PR	3500	Piglet production	Positive	635
C	MG	1000	Farrow-to-finish	Positive	760
D	MT	15,000	Two site herds *	Positive	880
E	RS	600	Two site herds *	Positive	745
F	GO	560	Farrow-to-finish	Positive	650
G	SC	2200	Two site herds *	Positive	825
H	ES	480	Farrow-to-finish	Negative	890
I	SP	150	Farrow-to-finish	Positive	520
J	MG	5200	Two site herds *	Positive	820
K	SP	540	Farrow-to-finish	Positive	615
L	SP	900	Two site herds *	Negative	685
M	DF	8000	Two site herds *	Positive	790
N	RS	300	Two site herds *	Negative	520
O	SC	1700	Two site herds *	Positive	550
P	MG	500	Farrow-to-finish	Positive	560
Q	MG	800	Farrow-to-finish	Positive	580
R	PR	1550	Farrow-to-finish	Positive	735
S	SP	3500	Farrow-to-finish	Positive	715
T	RS	600	Two site herds *	Positive	630
U	MG	1000	Farrow-to-finish	Positive	435
V	PR	480	Two site herds *	Positive	580
W	PR	2350	Farrow-to-finish	Positive	620
X	PR	5500	Two site herds *	Negative	1105
Y	MG	1500	Farrow-to-finish	Positive	860

* Piglet production unit and finishing herds (with animals in two different sites). DF—Distrito Federal, ES—Espírito Santo, GO—Goiás, MG—Minas Gerais, MT—Mato Grosso, PR—Paraná, RS—Rio Grande do Sul, SC—Santa Catarina, SP—São Paulo.

**Table 2:** Amount of antimicrobial used, the number of different drugs used, and percentage of pig lifetime submitted to a preventive antimicrobial use in evaluated herds.

Herd	M0				M1			
	mg ATB/Kg	Antimicrobials Drugs Used	% Pig Life Medicated	Productivity (Kg Pig/Sow/Year)	mg ATB/Kg ^a^	Antimicrobials Drugs Used ^b^	% Pig Life Medicated ^c^	Productivity (Kg Pig/Sow/Year) ^d^
A	344.3	8	90.4	2970	241.6	5	51.6	3950
B	532.3	8	73.7	2856	-	-	-	-
C	322.3	6	46.1	2754	111.0	4	21.5	2692
D	345.1	6	86.9	2270	646.3	8	87.2	3484
E	292.5	4	59.5	2870	-	-	-	-
F	330.3	7	53.3	3059	-	-	-	-
G	236.7	7	81.8	2835	160.0	5	29.8	2858
H	521.4	8	85.6	3071	30.2	2	18.5	3179
I	531.4	10	86.0	2004	-	-	-	-
J	372.1	9	84.0	2734	310.3	4	37.3	3642
K	573.4	9	84.4	2708	-	-	-	-
L	5.4	3	5.0	3979	5.4	3	5.0	4260
M	283.5	7	82.1	3013	-	-	-	-
N	27.6	2	2.9	2625	-	-	-	-
O	388.5	5	69.2	2530	-	-	-	-
P	247.2	6	60.0	2767	269.7	6	70.7	2849
Q	344.8	5	68.8	2825	257.6	3	37.5	2807
R	502.7	10	85.9	2510	-	-	-	-
S	488.3	8	87.5	3201	-	-	-	-
T	423.4	6	53.7	3422	271.8	8	67.8	2972
U	332.1	6	54.2	2402	232.0	5	30.5	2789
V	370.2	8	55.3	3342	-	-	-	-
W	345.8	9	83.7	2326	179.8	6	76.7	3663
X	212.9	4	30.5	3002	-	-	-	-
Y	585.6	11	87.7	3224	395.1	6	42.9	3700

^a^ Comparison by Wilcoxon test for paired samples, *p* = 0.034. ^b^ Comparison by Wilcoxon test for paired samples, *p* = 0.025. ^c^ Comparison by Wilcoxon test for paired samples, *p* = 0.015. ^d^ Comparison by Wilcoxon test for paired samples, *p* = 0.019.

**Table 3:** List of antimicrobials used in each production phase in the preventive/metaphylactic programs.

Herd	Farrowing	Nursery	Finisher
A	CEF *	AMO, COL, FLO, LIN/SPE, TIA	AMO, AVI, ENR, FLO, TIA
B	CEF, BMD	AMO, COL, DOX, LIN/SPE, TIA	AMO, DOX, TIA
C	AMO, TUL	AMO, COL, DOX, JOS	AMO, DOX, TIA
D	-	AMO, FLO, HAL	AVI, OXI, TIA
E	-	FLO	AMO, DOX, FLO, TIA
F	CEF	AMO, LIN, TIA	BAC, DOX, TIA, TIL
G	CEF	AMO, LIN, NOR	FLO, FLA, LIN/SPE, TIL
H	AMO, GEN	AMO, FLO, FOS, JOS	CLO, FLO, LIN, NOR
I	-	AMO, CLO, HAL, LIN, SUT	CLO, DOX, ENR, TIA
J	LIN/SPE	AMO, CLO, FLO	BAC, COL, DOX, TIA, TIL
K	AMO, GEN	AMO, CLO, DOX, JOS, TIA	AMO, DOX, ENR, FLO, NOR
L	LIN/SPE	AMO	-
M	CEF	AMO, CLO, DOX, FLO, TIA	DOX, ENR, TIA
N	-	-	AMO, TIA
O	-	AMO, CLO	DOX, FLO, TIA
P	-	AMO, TIA	DOX, FLO, LIN, TIA, TIL
Q	AMO	AMO, FLO, TIA	DOX, ENR, FLO, TIA
R	AMO/GEN	AMO, CLO, LIN/SPE, TIA, TIL	DOX, ENR, FLO, TIA, TIL
S	CEF	AMO, CLO, FLO, LIN	AMO, DOX, ENR, TIA
T	CEF	AMO, DOX, JOS, TIA	AMO, DOX, FLO, TIA
U	AMO/GEN	AMO, CLO, TIA	DOX, TIA, TIL
V	CEF	AMO, CLO, DOX, LIN/SPE, TIA	AMO, FLO, LIN, TIA
W	CEF	AMO, DOX, FLO, NOR, NEO, TIA	AMO, DOX, ENR, FLO, NOR, TIA, TIL
X	-	AMO, GEN, LIN	AMO, TIA
Y	CEF, TUL	AMO, COL, NOR, NEO, TIA	BAC, COL, DOX, FLO, TIA, TIL

* AMO−amoxicillin, AVI−avilamycin, BMD−bacitracin methylene-disalicylate, BAC−zinc bacitracin, COL−colistin, CEF−ceftiofur, CLO−chlortetracycline, DOX−doxycycline, ENR−enramycin, SPE−spectinomycin, FLA−flavomycin, FLO−florfenicol, FOS−fosfomycin, GEN−gentamicin, HAL−halquinol, JOS−josamycin, LIN−lincomycin, NOR−norfloxacin, NEO−neomycin, OXI−oxytetracycline, SUT−trimethoprim-sulfamethoxazole, TIA−tiamulin, TIL−tylosin, TILM−tilmicosin, TUL−tulathromycin.

## Data Availability

The data that support the findings of this study are available from the corresponding author upon reasonable request.

## Supplementary Materials

The following are available online at https://www.mdpi.com/article/10.3390/microorganisms9040881/s1, Table S1: Biosecurity questionnaire; Table S2: Measures to reduce antimicrobial use; Figure S1: Productivity parameters according to antimicrobial amounts used in 25 herds at M0; Figure S2: Amount of antimicrobials used per kg of pig produced and productivity index in 13 herds evaluated at M0 and M1, after the implementation of a good practices management program.
