# Supplementary material for: Antimicrobial Use in Brazilian Swine Herds: Assessment of Use and Reduction Examples
Source: Microorganisms. 2021 Apr 20;9(4):881. doi: 10.3390/microorganisms9040881 (PMC8074920; doi:10.3390/microorganisms9040881)
Supplement: Supplementary file 1 [file microorganisms-09-00881-s001.zip › Supl_Mat_Table S1.pdf]

Table S1: Biosecurity questionnaire

BIOSECURITY QUESTIONNAIRE

FARM NAME: **AUDITOR:**

FARM CHARACTERISTICS: **DATE:**

SITE CHARACTERISTICS:

COMMENTS:

ASPECT	Adequate	Need adjust	Inappropriate
**EDUCATION**	**10**	**5**	**0**
Presence of Biosecurity Program			
Presence of SOP related with Biosecurity			
Perform Biosecurity Audit (Frequency)			
Training of Employees			
**SITE**			
Pig Farm Proximity (< 3.0 km distance)			
Farms positive to FMD, PRV, CSF, PRRS			
Slaughterhouse Proximity (< 3.0 km distance)			
Pig Farm Density (< 1000 heads / 1000-5000 / > 5000 heads)			
Road Traffic (light / medium / high)			
Presence of other animals around the farm (goat, sheep, cattle)			
Presence of other animals inside the farm (dog, cat)			
Presence of "Green Belt"			
Presence and maintenance of fences			
Presence of Biosecurity Signs			
Policy of Entrance Gates			
**PERSONNEL**			
Visitor Book			
Downtime policy			

Employees are allowed to raise their own animals			
Shower-in and shower-out policy			
Cleaning and maintenance of shower area			
Stablished clean and dirty lines			
"Danish System" (no shower, just coveralls, boots and wash hands)			
Pesonal belongings are allowed inside the farm			
Clothing and boots provided			
Pork meat allowed in the site			
Contact or cross-traffic between drivers and farm employees during load procedures			
**ISOLATION OF INCOMING ANIMALS**			
Distance of Isolation facilities from barns (> 500 meters)			
Isolation period > 30 days			
Shower-in and shower-out policy			
Personnel movement			
Transport to Isolation			
Testing procedures			
ELISA tests performed (PRRS, *Mycoplasma*,…)			
PCR tests performed (PRRS, *Mycoplasma*,CSF, PRV…)			
Health Status of Incoming Animals			
Source of Incoming Animals (more than one)			
Frequency of Incoming Animals			
Transported on dedicated trailers			

**EQUIPMENT / SUPPLIES**			
UV light or Disinfection Chamber present			
UV light or Disinfection Chamber properly operated			
Equipment/Supplies shared between farms			
Frequency of supply delivery			
Supply delivery follow health pyramid			
Supply delivered in dedicated trailers			
**PEST CONTROL**			
Rodent control			
Bait stations every 15-20 meters			
Bait stations filled			
Bait log current			
Insect (cockroaches, flies) control			
Bird proof			
Feed bins lids closed			
Feed spills are being cleaned-up			
Maintenance of area around the barns (grass/weeds/garbage)			
Standing water drained around the farm			
**SEMEN**			
Produced in the same site			
Delivered to separate location			
Semen is being delivered via the double bag technique			
Sanitary monitory frequency (Which diseases?)			
**WATER**			
Water source (deep well, shallow well, surface)			
Water storage			
Water treatment (chorine, hydrogen peroxide)			
System cleanup frequency			
Water analysis frequency			

**AIR**			
Filtered farm (management of filters)			
**ANIMAL TRANSPORT**			
Drivers have received biosecurity training			
Drivers have used clean coverall, boots and gloves for each delivery			
Drivers raise their own pigs			
Trucks and trailers have been properly cleaned, washed, disinfected and dried			
Detergent used (product, concentration, dilution)			
Disinfectant used (product, concentration, dilution)			
Truck / Trailer downtime followed			
Delivery follow health status of sites			
Transport log completed			
Truck / Trailer swabbing (frequency)			
Lines of separation are observed in loading chutes area			
Loading chutes are being cleaned and disinfected prior and after each use (and prior to receive stock)			
**INTERNAL TRANSPORT (GILTS OR CULLS)**			
Drivers have received biosecurity training			
Transport log completed			
Cleaning and disinfection procedure (frequency, product, concentration, dilution)			
Trailer stored or covered			
**FEED DELIVERY TRANSPORT**			
Drivers have received biosecurity training			
Drivers raise their own pigs			
Delivery outside fence			
Plastic boots utilized			
Bag delivery acceptable			
Dedicated trucks and trailers			
Truck / Trailer downtime followed			

Delivery follow health status of sites			
Transport log completed			
Cleaning and disinfection procedure (frequency, product, concentration, dilution)			
**GENERAL MANAGEMENTS**			
Cleaning / Disinfection of tools used during piglets processing			
Wash hands between litters			
Boot bath management			
Cleaning and disinfection procedure in Farrowing (frequency, product, concentration, dilution)			
Cleaning and disinfection procedure in Nursery and Finishers (frequency, product, concentration, dilution)			
Respect downtime of facilities between batches			
Adopt all-in all-out procedure			
Frequency of needles change during main herd vaccination			
Frequency of needles change during main herd medication			
Frequency of needles change during piglets vaccination			
Frequency of needles change during piglets medication			
Vaccination program			
Quality control of vaccines			
Medicines storage			
Medicines in use (injectable, water and feed)			
Feed bins maintenance			
Electrical meter reading - outside perimeter			
**DEAD DISPOSAL**			
Removal frequency			
Only designated area is used to remove mortality and it's properly cleaned and disinfected after use			
Physical contact back into the farm from tractor, dead cart, buckets, clothing or other equipment used for mortality removal			
Equipment cleaned and disinfected each time it goes to the dumpster / cooler			

Farm staff do not re-enter the farm after completing dead run			
System properly used (Incineration / Compost / Rendering / Pit)			
**GARBAGE PICK-UP**			
Frequency			
Dumpster location outside			
**EFFLUENT MANAGEMENT**			
Reponsible personnel have received biosecurity training			
System properly used (Pit / Lagoon / Biogas)			
Gutter flush			
Presence of erosion			
Effluent distribution (field or other system)			
TOTAL	0	0	0

SCORE SYSTEM	
PONTUATION	RISK LEVEL
**0-300**	Extremely high risk
**301-600**	Really high risk
**601-900**	High risk
**901-1000**	Medium high risk
**1001-1100**	Medium risk
**1101-1200**	Low risk
