# Supplementary material for: Antimicrobial Use in Brazilian Swine Herds: Assessment of Use and Reduction Examples
Source: Microorganisms. 2021 Apr 20;9(4):881. doi: 10.3390/microorganisms9040881 (PMC8074920; doi:10.3390/microorganisms9040881)
Supplement: Supplementary file 1 [file microorganisms-09-00881-s001.zip › Supl_Mat_Table S2.pdf]

Table S2: Measures to reduce antimicrobial use.

THE FOLLOWING ACTIONS OF GOOD PRACTICES WERE PERFORMED AT HERDS:

a) Optimize sows milk production through adjustments in their diets, as well adjustment in feed program reaching proper sows body condition
b) Increase level of nutraceutical ingredients in Nursery and Finisher diets as plasma protein, replacing AGP by probiotic (mainly Lactobacillus spp. based), essential oils and blend of organic acids.
c) Provide blend of organic acids through water during first three weeks after weaning bringing water pH to 3,5 avoiding proliferation of *Enterobacteriaceae*.
d) Provide management adjustments like improvement of navel disinfection procedure, stop preventive use of antimicrobial in farrowing, replacing it by probiotic/prebiotic use, minimize cross-fostering, adjust weaning age (minimum 21 days, average close to 24-25 days), adjust female replacement rate (minimum 40,0%/ year), update cleaning and disinfection procedure in empty rooms, as well in the presence of animals, realization of periodical cleaning and disinfection of water tank.
e) Stablish environmental adjustments like improve heating in nursery facilities, change nursery floor from partial compact to completed slatted, provide plastic curtain in growing finishing rooms.
f) Propose cultural changes about antimicrobial use: Stimulate the commitment from owner and employees doing training and awareness about biosecurity policies, good practices, and implications of antimicrobial rational use.
g) Improve the proper diagnostic of sanitary challenges presents at the system doing material collection from different phases and evaluating animals at slaughterhouse to measure impact of respiratory diseases on animal performance.
h) Update the vaccination program introducing new vaccines if necessary, according to challenge.
i) Provide corrections in weak points on biosecurity policies and procedures updating them.
