# Supplementary figures and images for: Antimicrobial Use in Brazilian Swine Herds: Assessment of Use and Reduction Examples
Source: Microorganisms. 2021 Apr 20;9(4):881. doi: 10.3390/microorganisms9040881 (PMC8074920; doi:10.3390/microorganisms9040881)

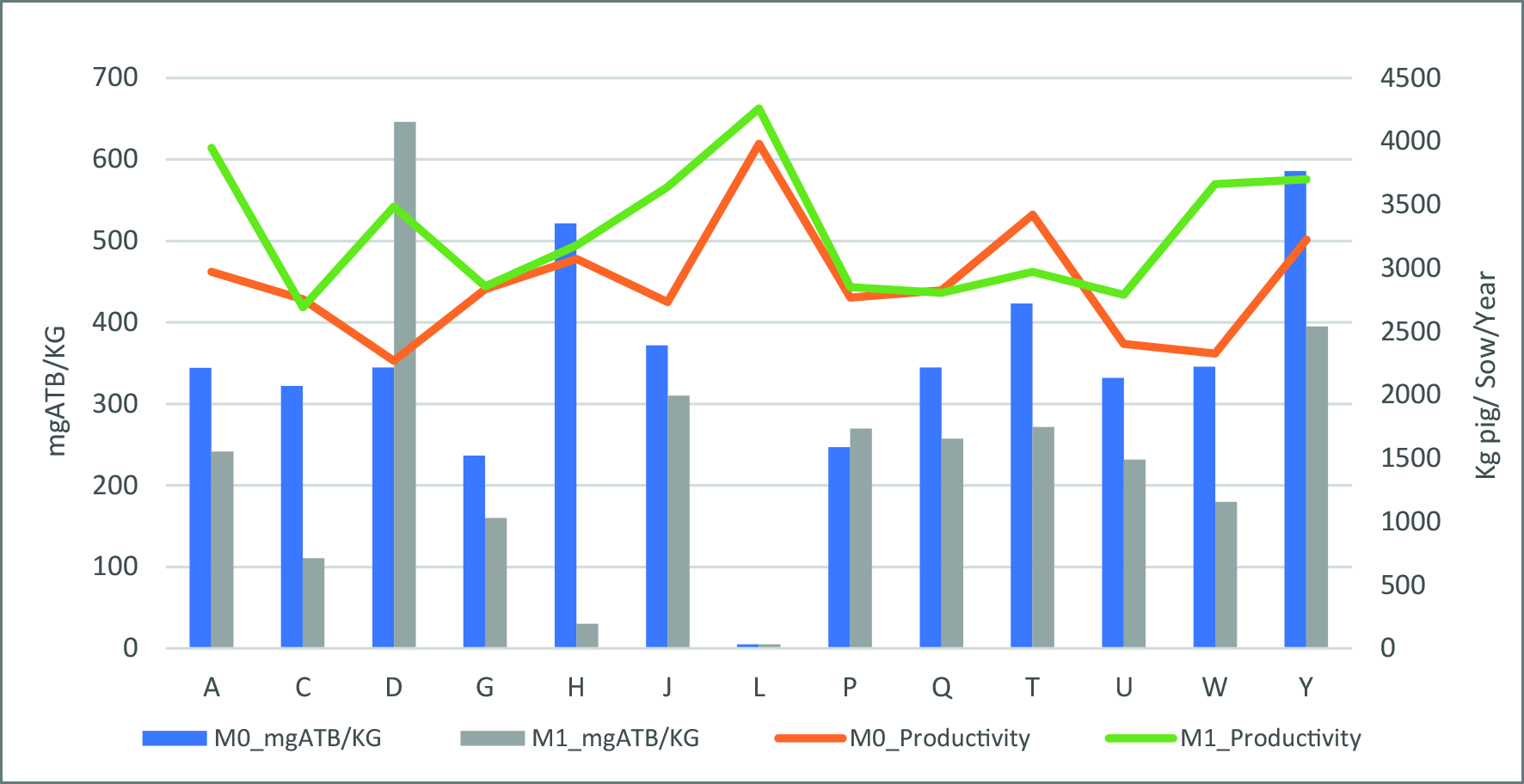

Supplement: Supplementary file 1 [file microorganisms-09-00881-s001.zip › Figure S2.tif]

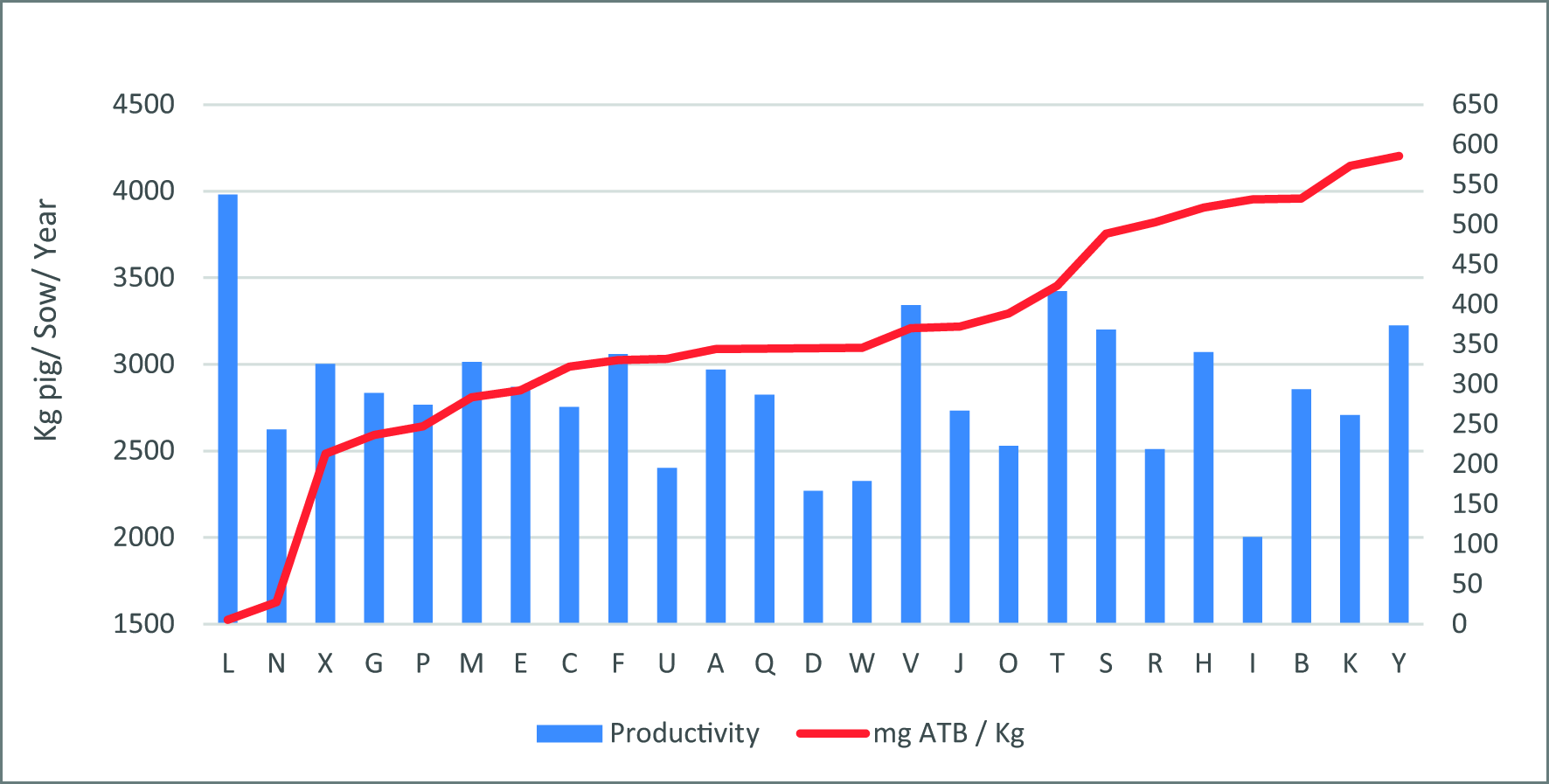

Supplement: Supplementary file 1 [file microorganisms-09-00881-s001.zip › Figure S1.tif]
